# The Clinical Incorporation of the 2017 Classification of Periodontal Disease Conditions Part II: The Association Between Clinical Parameters and Gender

**DOI:** 10.7759/cureus.63737

**Published:** 2024-07-03

**Authors:** Ashwini Athul, Priyanka TG, Nasser I, Sangavi G, Nagaland Tirupati, Agila Elumalai, Shanmugam Muthukali, Ashwath Balachandran

**Affiliations:** 1 Periodontics, Chettinad Dental College and Research Institute, Chennai, IND; 2 Public Health Dentistry, Chettinad Dental College and Research Institute, Chennai, IND

**Keywords:** chronic gingivitis, periodontitis, periodontal diseases, gender, classification

## Abstract

Background

The implementation of the new classification system guided clinicians in the best way for disease diagnosis and patient management. The advent of definitions such as clinical gingival health additionally helped in distinguishing between intact and reduced periodontium. The purpose behind introducing new systems was to guide practitioners to improve on managing the requirements of the patients effectively. It also enables professionals to concentrate on clinical practice, and it can be suggested that readers who are concentrating on research make use of the global classification system. In this study, we present a diagnostic approach for periodontal diseases that supports the innovative classification system in relation to gender while remaining compatible with previous guidelines.

Aim

The present study focuses on the relationship between gender association and the clinical parameters in periodontitis patients that would support the new classification system.

Materials and methods

This was a retrospective, cross-sectional, observational study consisting of patient data obtained from past patient records who were diagnosed with periodontitis.

Results

The data analysis results showed that 95% of the population was similarly distributed across the genders in terms of frequency, and there was minimal statistically significant difference between the genders when compared to the clinical parameters. However, there was a statistically significant difference in the specific clinical characteristics, such as recession, which impacted 48.6% of male patients, and the presence of local factors, which affected 43.2% of female patients, in addition to the 59.4% localized involvement of periodontitis. All the data were analyzed using the SPSS software version 20 (IBM SPSS Statistics, Armonk, NY). The chi-square test was used to evaluate the descriptive analysis and frequency tables.

Conclusion

The addition of correlating clinical parameters with data based on gender had no effect on the new classification system. Meanwhile, it can be used as an adjunct to evaluate the distribution and extent of disease progression among genders.

## Introduction

The polymicrobial disease of the oral cavity, such as periodontitis, results in crucial damage to the teeth involved, when not diagnosed and treated appropriately [1]. Periodontal disease has many variants, which led to numerous classification systems over time, in order to define different pathological conditions and to enhance the clinical, research-based, therapeutic, and epidemiological factors [2,3]. Depending on the extent, rate of progression, severity, and localization of periodontal diseases, came the 1999 periodontal classification system given by the International Workshop for the Classification of Periodontal Diseases and Conditions. Later in 2017, the American Academy of Periodontology (AAP) and the European Federation of Periodontology (EFP) established the updated definitions of diseases associated with gingivitis and periodontitis, especially highlighting both health and disease conditions, thus making a precise diagnosis with staging and grading systems for the clinicians to efficiently provide professional oral health care to the patients [4].

The main goal of obtaining a new classification system is to reduce the complex patient data previously acquired and simplify it so as to easily make the clinician identify the disease pattern as early as possible. With the basic periodontal examination (BPE) introduced, one can assess the treatment needs of a patient. However, it does not provide the diagnosis or predicted prognosis after treatment. The current status of the patient is achieved especially for those patients who were previously affected by periodontitis. Although they are completely treated with appropriate therapy, they are still considered to have periodontitis as they remain in a maintenance period throughout their life. They seem healthy but with reduced periodontium, where they are associated with either periodontitis or non-periodontitis etiology [5]. Probing depth (PD), attachment loss, gingival recession, furcation involvement, bleeding on probing (BOP), tooth mobility, tooth loss, extent of disease, index systems, and periodontal screening and recording (PSR) have all emerged as the clinical parameters to be assessed, out of which the probing depth and BOP play a crucial role in determining the health and disease of the teeth involved. The BOP of <10% and the PD of <3 mm with the absence of attachment loss indicate gingival health with intact periodontium and with the presence of attachment loss indicate reduced periodontium.

Classification systems being diverse in periodontal diseases are still under development and aim to reduce the complexity. The present study is the continuation of the study correlating the diagnosis of periodontitis involving 4993 patients using the new classification system, with the aim of incorporating gender-based associations with clinical features and risk variables.

The gender-based association has been a rare entity in the new classification system. It has also been demonstrated that there are gender differences in the occurrence of periodontitis, with males generally being diagnosed with the disease at a higher rate than females. It is found that females tend to seek oral care more than males in terms of hygiene and aesthetics. However, with the hormonal changes occurring during puberty and pregnancy, menopausal females tend to have more gingival and periodontal clinical manifestations [6]. The primary objective of this retrospective study is to determine if there is any effect in the new classification system when associated with gender and clinical parameters. Also, the secondary objective is to evaluate whether the results can streamline the classification system and be applied in clinical practice.

This study focuses on the relevance of using the new classification system in routine dental practice, which will improve communication among dentists, allowing for appropriate diagnosis and comprehensive treatment planning.

## Materials and methods

Study design

Retrospective, cross-sectional, noninterventional research was the design of this project. Every subject that satisfied the inclusion and exclusion criteria was split into male and female categories according to gender. The 1999 American Academy of Periodontology (AAP) Classification of Periodontal Diseases and the 2017 AAP Classification of Periodontal Diseases and Conditions were the diagnostics used for correlating with gender in addition to the clinical features of periodontal diseases and index systems [2,3,5].

Sample size calculation

After performing a goodness-of-fit test using sample sizes from comparable research, the minimum sample size needed for this investigation was determined to be 4800, for which the study's power was 0.95. As a result, we preferred to include 200 more samples, which was more than the minimal number needed in order to improve the study's validity. Therefore, the sample size increased up to 5000. However, six patients deferred from the study as they were not diagnosed with periodontitis, and one patient was under 18 years of age. Hence, a total of 4993 patients were included in the study.

The statistical analysis was performed using the SPSS software version 20 (IBM SPSS Statistics, Armonk, NY). Using the chi-square test, a descriptive analysis and frequency tables were presented, and the relationship between the clinical parameters and gender was evaluated.

Subject population and selection

The present study was approved by the Institutional Human Ethical Committee of Chettinad Academy of Research and Education, Kelambakkam (approval number: IHEC-II/0305/23). A single dental practitioner from the department of periodontology at the Chettinad Dental College and Research Institute collected all the participant data retrospectively using Excel software (Microsoft Corp., Redmond, WA). The collecting of this data took place between October 2019 and April 2024. The department of public health dentistry at Chettinad Dental College and Research Institute calculated the sample size and conducted the analysis and interpretation of the data. The inclusion criteria were systemically healthy individuals of ages between 18 and 55 years and those patients who were diagnosed with gingivitis and periodontitis. Gender, diagnostic information, and clinical characteristics such as recession, furcation involvement, tooth loss, and tooth mobility were taken into consideration when obtaining the data. Additionally, periodontal screening and recording with index systems such as Russell's Periodontal Index (PI) and the Simplified Oral Hygiene Index (OHI-S) were assessed. The study excluded patients with systemic conditions such as uncontrolled diabetes, heart disease, liver disease, and blood disorders; patients using immunosuppressant, alcohol, and tobacco; and patients who are pregnant or lactating.

## Results

A total of 4993 patients' details were acquired, comprising 2715 (54.4%) male and 2278 (45.6%) female individuals. The remaining six patients were excluded from the group since they were not diagnosed with periodontitis. With a standard deviation of 12.5, the average age was 31.9 years. Table 1 shows the frequency distribution of the different clinical symptoms.

**Table 1:** Frequency distribution and mean percentage of clinical parameters DBIG, dental biofilm-induced gingivitis; OHI-S, Simplified Oral Hygiene Index; PSR, periodontal screening and recording; PI, periodontal index

Variable	Description	Frequency	Percent
Tooth loss	No tooth loss	4077	81.6
	Caries	520	10.4
	Due to periodontitis	299	6.0
	Other reasons	98	2.0
Recession	No recession	2369	47.4
	Class I	2037	40.8
	Class II	361	7.2
	Class III	179	3.6
	Class IV	48	1.0
Mobility	No mobility	4118	82.5
	Grade I	664	13.3
	Grade II	152	3.0
	Grade III	60	1.2
Furcation	No furcation	4925	98.6
	Grade I	29	0.6
	Grade II	40	0.8
Extent	Localized periodontitis	2834	56.7
	Generalized periodontitis	223	4.5
	Molar/incisor pattern	119	2.4
	Generalized gingivitis	1223	24.5
	Localized gingivitis	595	11.9
Diagnosis	Clinical gingival health	11	0.2
	DBIG, biofilm alone	2212	44.3
	DBIG, risk factors	172	3.4
	Stage 1, grade A periodontitis	1013	20.3
	Stage 2, grade B periodontitis	1032	20.7
	Stage 3, grade with periodontitis	66	1.3
	Stage 4, grade B periodontitis	488	9.8
OHI-S	Good	1158	23.2
	Fair	3375	67.6
	Poor	461	9.2
PSR	Healthy	83	1.7
	Bleeding after probing	1507	30.2
	Supra/subgingival calculus	1994	39.9
	Shallow pocket	953	19.1
	Deep pocket	442	8.9
	Furcation	15	0.3
Russell's PI	Clinically normal	976	19.5
	Simple gingivitis	1911	38.3
	Beginning destructive periodontal disease	1686	33.8
	Established destructive periodontal disease	391	7.8
	Terminal disease	30	0.6

Based on the frequency distribution of different clinical symptoms, among the patients who were diagnosed with periodontitis, 299 (6%) revealed tooth loss that occurred due to periodontitis, 520 (10.4%) revealed tooth loss that occurred due to caries, 2037 (40.8%) showed class I recession, 664 (31.3%) showed grade I mobility, and 40 (0.8%) showed grade II furcation. Among the assessed patients, 2834 (56.7%) revealed localized periodontitis, 2212 (44.3%) were diagnosed with dental biofilm-induced gingivitis, and 1032 (20.7%) were diagnosed with stage II grade B periodontitis. Three thousand three hundred seventy-five (67.6%) patients showed a fair interpretation of OHI-S, 1994 (39.9%) revealed supragingival and subgingival calculus, and 1911 (38.3) revealed simple gingivitis.

**Figure 1:**
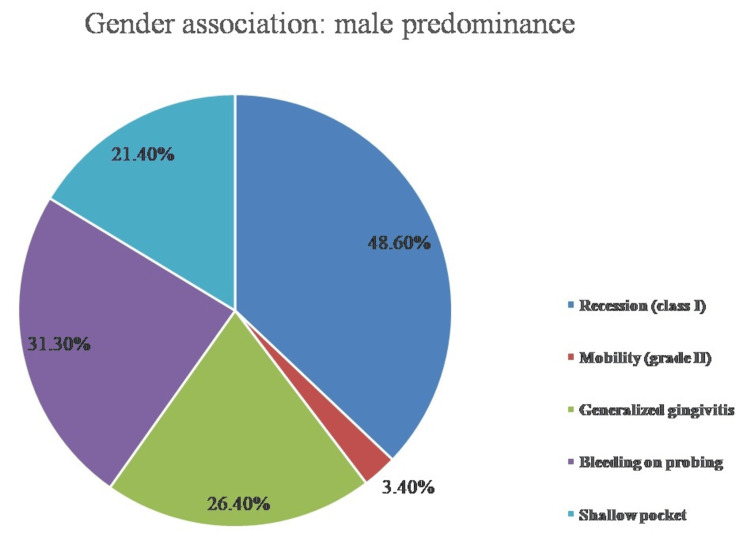
Gender association with clinical features predominant among the male population

Individual clinical features associated with gender among patients are interpreted in Table 2. Clinical characteristics including furcation involvement and tooth loss showed no statistically significant difference (p-values of 0.48 and 0.53, respectively). However, among the male population, a clinical trait called tooth mobility was discovered to be statistically significant with a p-value of 0.036. Figure 1 interprets the gender association with clinical characteristics that are predominant among the male group.

**Table 2:** Features of periodontitis associated with gender Statistically significant results are marked in bold (p-value<0.05) DBIG, dental biofilm-induced gingivitis; OHI-S, Simplified Oral Hygiene Index; PSR, periodontal screening and recording; PI, periodontal index; n, number of patients in each group

Variable	Description	Male		Female		P-value
		n	%	n	%	
Tooth loss	No tooth loss	2216	81.6	1861	81.7	0.53
	Caries	279	10.3	235	10.3	
	Due to periodontitis	168	6.2	131	5.8	
	Other reasons	52	1.9	45	2.0	
Recession	No recession	1258	46.3	1111	48.7	0.001
	Class I	1129	41.6	908	39.8	
	Class II	202	7.4	159	7.0	
	Class III	114	4.2	65	2.9	
	Class IV	12	0.4	36	1.6	
Mobility	No mobility	2239	82.5	1879	82.4	0.036
	Grade I	361	13.3	303	13.3	
	Grade II	92	3.4	60	2.6	
	Grade III	23	0.8	37	1.6	
Furcation	No furcation	2681	98.7	2244	98.5	0.489
	Grade I	16	0.6	13	0.6	
	Grade II	18	0.7	22	1.0	
Extent	Localized periodontitis	1481	54.5	1353	59.4	0.001
	Generalized periodontitis	131	4.8	92	4.0	
	Molar/incisor pattern	73	2.7	46	2.0	
	Generalized gingivitis	718	26.4	505	22.2	
	Localized gingivitis	312	11.5	283	12.4	
Diagnosis	Clinical gingival health	6	0.2	5	0.2	0.006
	DBIG, biofilm alone	1147	42.2	1065	46.7	
	DBIG, risk factors	103	3.8	69	3.0	
	Stage 1, grade A periodontitis	551	20.3	462	20.3	
	Stage 2, grade B periodontitis	593	21.8	439	19.3	
	Stage 3, grade with periodontitis	29	1.1	37	1.6	
	Stage 4, grade B periodontitis	286	10.5	202	8.9	
OHI-S	Good	610	22.5	548	24.0	0.412
	Fair	1850	68.1	1525	66.9	
	Poor	255	9.4	206	9.0	
PSR	Healthy	45	1.7	38	1.7	0.001
	Bleeding after probing	851	31.3	656	28.8	
	Supra/subgingival calculus	1009	37.2	985	43.2	
	Shallow pocket	581	21.4	372	16.3	
	Deep pocket	221	8.1	221	9.7	
	Furcation	8	0.3	7	0.3	
Russell's PI	Clinically normal	528	19.4	448	19.7	0.717
	Simple gingivitis	1054	38.8	857	37.6	
	Beginning destructive periodontal disease	908	33.4	778	34.1	
	Established destructive periodontal disease	206	7.6	185	8.1	
	Terminal disease	19	0.7	11	0.5	

The index systems Simplified Oral Hygiene Index (OHI-S) and Russell's Periodontal Index (PI) also had no statistically significant difference with p-value of 0.41 and 0.7, respectively.

A statistically significant result with a p-value of 0.006 was obtained when the diagnosis was interpreted in relation to gender and was discovered predominant among the female population.

**Figure 2:**
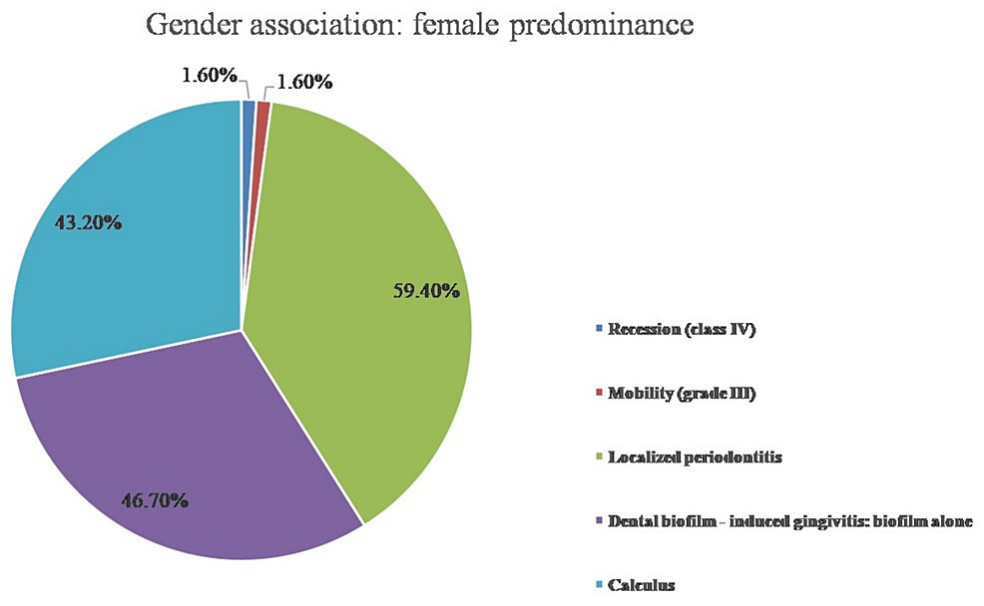
Gender association with clinical features predominant among the female population

In addition, the male population had a considerably higher frequency of class II and class III gingival recession, bleeding on probing, and shallow pockets, all of which were associated with a p-value of 0.001. The degree of localized periodontitis and the presence of supra- and subgingival calculus, both of which exhibited statistically significant differential results with a p-value of 0.001, were the parameters that favored the female population. Figure 2 interprets the gender association with clinical characteristics that are more common in the female population. The chi-square test was used to statistically analyze each of these parameters among patients with periodontitis.

## Discussion

The goal of the current study was to incorporate a gender-based relationship with the clinical features of individuals with periodontitis. The aim was to disseminate the data to the clinicians so that they could integrate them into their standard clinical practice and align with the 2017 classification. When compared to the clinical features, the results of the current investigation showed that 95% of the population had results that were evenly distributed across genders.

Such a huge participation of patients was possible due to periodontal recall to the patients by the respected practitioner and also the patient compliance, which were highly demanding in such situations. Regular dental visits incorporating routine clinical examination, mechanical debridement, and reinforcement of oral hygiene instructions encouraged the patients to regularly visit the dental clinics and hospitals.

According to the current study, there were 54.4% of males and 45.6% of females in the population. The remaining people did not meet the inclusion criteria as they had not been diagnosed with periodontal disease. These findings corroborated the findings of research by Benoist et al. [7], which showed a 53% male predominance. Ravidà et al. [8] and Graetz et al. [9] found that despite the literature indicating a higher proportion of male predominance, a study with a female population of 52.1% and 60.2%, respectively, demonstrated a higher female predominance.

Gingival recession was found to be more common in the male population when examining the clinical characteristics of periodontitis in relation to gender. In the male population, class II gingival recession was 7.4%, and class III gingival recession was 4.2%. This male predominance has been shown to be in accordance with the research conducted by Arowojolu [10] and Toker and Ozdemir [11].

With the advent of the 2017 classification system, a new definition for periodontal health emerged with two clinical scenarios, namely, clinical gingival health on the intact periodontium and clinical gingival health on the reduced periodontium. The difference between these two types is the presence of attachment loss, which is elucidated as a reduced periodontium due to non-periodontitis reason. The present study stated that the disease occurrence in patients who were affected with periodontitis was associated with significant attachment loss.

Localized periodontitis as a means of depicting the extent of the severity of the disease greatly influenced the female population more than males in the present study. This female predominance was found to be in accordance with a study performed to assess the prevalence of chronic periodontitis exclusively for the female population in Saudi Arabia. Studies by Boillot et al. [12] and Burt [13] that show the levels of education and occupation, the concern for oral health, and the frequency of dental appointments have clearly shown the imbalance in socioeconomic position. The deficiency in all of these conditions led to the female population having a high chance of acquiring the disease with 12% of localized periodontitis and 14% of generalized periodontitis. These results were found to be consistent with the periodontal screening and recording feature, which indicated that females were more likely than males to have local factors such as supra- and subgingival calculus. However, a study found that, regardless of the amount of calculus or soft plaque deposits, males often had worse oral hygiene than females [14].

In the present study, bleeding on probing values is found to be higher in the male population. This was found in connection with a study conducted by Shiau and Reynolds [15], which proposed that pockets and bleeding, coupled with clinical attachment loss of all severity levels, are often more common in males than in females.

Additionally, in the prevalence research of dental calculus among adults, Beiswanger et al. [16] reported that, among the 1426 participants in the study, more males had calculus than females. Nonetheless, there was a direct correlation between the two genders' calculus formation regardless of age.

According to the current study, males' tooth mobility differs from females' by 3.4%. A prevalence investigation carried out in a tertiary health-care center, in contrast to this study, revealed that females have a higher rate of tooth mobility than males. However, Giannakoura et al. [17] and Azodo and Ogbebor [18] claimed to have hormonal problems in common for both genders. An overall analysis of the population showed a greater prevalence of males, particularly among Indians [19].

The current study demonstrates that periodontal destruction is more caused in males than in females. This difference in oral health was likely due to worse oral cleanliness, negative attitudes toward oral health, and dental visit behavior among males, rather than genetic factors. According to the epidemiology statistics from the National Health and Nutrition Examination Survey, males developed periodontal diseases at a higher rate than females (56.4% versus 38.4%). Other studies also have consistently found a higher frequency in males [20].

Meanwhile, the British Society of Periodontology (BSP) developed the basic periodontal examination (BPE) in 1986. This examination included codes designed to evaluate the clinical condition of each patient's oral health. The fundamental ones were codes 0, 1, and 2. The existence of supra- and subgingival calculus was indicated by code 2. Codes 3 and 4 detailed the patient's loss of attachment and the necessity for more care. Thus, BPE offers fundamental guidance for the patient's therapy needs.

In general, males had the tendency to have periodontal disease greater than females. Also, regarding the progression of disease, they do not seem to be affected more rapidly than females. The present study correlated the diagnosis-based classification, namely, the dental biofilm-induced gingivitis and localized periodontitis attributed toward the female population. The cause may be due to hormonal imbalances and financial barriers that they may not adhere to the treatment protocols. Yet, it is more often that females prefer to have regular dental visits more than males.

The etiology behind these gender differences is still unclear and can be related to various confounding factors such as demographic, genetic, biological, or epigenetic [21,22]. The exploratory case study provided currently indicated age as an associated factor for moderate to severe periodontitis cases, but it was unable to distinguish gender, which was the main variable being investigated as an apparent factor of risk, due to limited statistical strength and study drawbacks such as retrospective study design. This was supported in the literature quoted by Natto et al. [23] stating age as the most prevalent confounding risk factor, followed by gender and race with respect to periodontitis. Furthermore, to make the relationship between gender and periodontal disease more compatible with the new classification systems, more research is needed to fully comprehend this relationship [24].

Study limitation

There was a disparity in the number of male and female subjects recruited for the current investigation. Thus, additional investigations were needed to validate the findings by recruiting equal numbers of male and female subjects. The study population incorporated a wide range of age groups because of which age was not considered as the primary risk variable. The study was conducted for a limited number of years, which affects the long-term follow-up, and the patients under supportive periodontal therapy have to be reviewed further to assess their periodontal status. Furthermore, the study was employed from prior patient records in order to assess the versatility, credibility, and viability in utilizing the new 2017 classification system in general health-care settings.

## Conclusions

In summary, it can be depicted from the above study that the male population is more prone to have periodontal diseases than the female population. The male gender was found to act as an independent risk factor for various clinical features of periodontal diseases, and the results were inclined toward the male population rather than the female population. The addition of gender-based criteria for association with clinical parameters appears to be a supplement to the 2017 classification system. There is still a lack of knowledge and comprehension regarding the exact role that gender plays, so more research is needed. The prevalence of a disease based on gender within a community cannot always be ascertained with total reliability.

Different risk factors affect the prevalence differently in the two genders. With this paradigm in place, research that is longitudinal in nature will be able to better understand the mechanisms underlying gender's function as a risk factor for periodontal disease and its impact on prevalence, progression, and severity. In the meantime, dental practitioners can ensure that the general population receives professional treatment by educating them with educational resources.
